# Editorial: The role of the basal ganglia in somatosensory-motor interactions: evidence from neurophysiology and behavior, volume II

**DOI:** 10.3389/fnhum.2023.1211465

**Published:** 2023-05-17

**Authors:** Martijn Beudel, Antonella Macerollo, Matt J. N. Brown, Robert Chen

**Affiliations:** ^1^Department of Neurology, Amsterdam UMC, Amsterdam Neuroscience, University of Amsterdam, Amsterdam, Netherlands; ^2^The Department of Pharmacology and Therapeutics, Institute of Systems, Molecular and Integrative Biology, University of Liverpool, Liverpool, United Kingdom; ^3^The Walton Centre NHS Foundation Trust, Liverpool, United Kingdom; ^4^Department of Kinesiology, California State University Sacramento, Sacramento, CA, United States; ^5^Division of Neurology, Department of Medicine, University of Toronto, Toronto, ON, Canada

**Keywords:** basal ganglia (BG), deep brain stimulation (DBS), micro electrode recording, local field potential (LFP), sensorimotor, traumatic brain injury (TBI), pain

In the second edition of the Research Topic, named “*The role of the basal ganglia in somatosensory-motor interactions: evidence from neurophysiology and behavior II*,” seven articles have been published. All seven contributions consisted of original work. Interestingly, each article used a unique approach (see Table 1) to study basal-ganglia function varying from psychophysics (Sengupta et al.) to invasive micro-electrode recordings (MER, Filyushkina et al.). This heterogeneity of approaches is in line with the approaches described in its predecessor (Beudel et al., 2020).

**Table 1:** Overview of the published articles.

**References**	**Type**	**(Brief) title**	**(Unique) technique**	**Disease**
Basha et al.	Original	Beta band oscillations in the motor thalamus	LFP	ET/PD
Campbell et al.	Original	The impact of pulse timing on cortical and subthalamic nucleus deep brain stimulation evoked potentials	EEG	PD
Chen et al.	Original	Disrupted Brain Structural Network Connection in *de novo* Parkinson's disease with RBD	DTI	(RBD) PD
Filyushkina et al.	Original	Attenuation of neural responses in subthalamic nucleus during internally guided voluntary movements in Parkinson's disease	MER	PD
Lan et al.	Original	Functional connectivity of the basal ganglia subregions in pain syndromes	Resting state fMRI	Pain syndrome
Pinky et al.	Original	Multimodal magnetic resonance imaging of youth sport-related concussion	Multimodal MRI	TBI
Sengupta et al.	Original	Exploration of sensory-motor tradeoff behavior in Parkinson's disease	Psychophysics	PD

LFP, local field potential; ET, essential tremor; PD, Parkinson's disease; EEG, electro-encephalography; RBD, rapid eye movement sleep behavior disorder; DTI, diffusion tensor imaging; MER, micro electrode recording; fMRI, functional magnetic resonance imaging; TBI, traumatic brain injury.

One important question is whether the approaches used in the current edition were available in the era of the first edition (2019–2020). Although 3 years seems a short period, as we know after the pandemic, a lot can happen. One important development in studying the basal ganglia over the last 3 years is the availability of commercially available DBS devices by at least three companies that can record local field potentials (LFP's) from fully implanted devices (Chen et al., 2020; Marceglia et al., 2022; Stam et al., 2023). However, despite these developments, initial explorations have mainly focused on the feasibility of using this new technique before research programs will adapt toward LFP recordings from fully implantable DBS devices. At this moment, certain limitations in this technology are still present. For example, sampling frequencies are still restricted to 256 Hz by some devices, which makes it impossible to perform the pulse-timing work at the millisecond scale by Campbell et al. and the MER work by Filyushkina et al.. In theory, the third study that made use of subcortical recordings and studied the beta band (13–30 Hz) in the thalamus (Basha et al.), could have made use of the currently available fully-implantable devices that have also been able to record LFP activity in the thalamus (Buijink et al., 2022). Since LFP recordings from fully-implantable devices offer many advantages such as the possibility to record for longer periods and to apply adaptive stimulation paradigms (Nakajima et al., 2021), it is expected that the field will adapt this technology in the coming years.

Next to the applied methods in the Research Topic, the diseases studied are also noteworthy. Although the role of the basal ganglia in movement disorders has been studied extensively, this is far less the case for (urologic) pain syndromes and traumatic brain injury (TBI) as respectively described by Lan et al. and Pinky et al.. The findings of the study by Lan et al. further illustrate the role of the basal ganglia in pathological somatosensory-motor interactions in abnormal sensations (i.e., pain) and Pinky et al. studied the role of the caudate as modulator in the recovery of TBI. These insights show how studying basal-ganglia functions beyond movement disorders can help in developing neuro-modulation strategies and the development of (image-based) biomarkers. The development of biomarkers is also relevant for the prodromal stages of Parkinson's disease (PD) such as in patients with rapid eye movement sleep behavior disorder (RBD), which are at a higher risk for developing PD. By identifying these patients using advanced imaging approaches, such as described by Chen et al., patients may benefit in the future from disease- modifying therapies before the onset of motor symptoms (Athauda et al., 2019).

Although these insights from imaging studies comparing patient populations with controls are valuable for the reasons mentioned above, they lack a behavioral component showing how altered (network) processing actually leads to disturbed basal-ganglia function. In their contribution Sengupta et al. show the nature of response disinhibition in PD during natural movement performance whilst (Filyushkina et al.) were able to distinguish neural patterns of self-initiated vs externally cued response in the STN. With the dawn of DBS stimulation paradigms that influence decision making processes (Ghahremani et al., 2018; Herz et al., 2018), it is of utmost relevance to understand the basal-ganglia contributions underlying autonomous action selecting and its interference by either diseases (e.g., PD) or therapies (e.g., DBS).

## Author contributions

All authors contributed to the editorial and have seen all revisions, including the final revision.
